# Assessing the impact of climate change on habitat dynamics of *Hovenia dulcis* in China using the MaxEnt model

**DOI:** 10.3389/fpls.2025.1641811

**Published:** 2025-10-21

**Authors:** Xi Li, Peiyao Li, Shimeng Li, Mingli Hu, Yankun Li, Yuanxin Li, Shi Wang, Ting Shu, Mingrong Yang, Qiqing Cheng

**Affiliations:** ^1^ School of Pharmacy, Xianning Medical College, Hubei University of Science and Technology, Xianning, Hubei, China; ^2^ Hubei Engineering Research Center of Traditional Chinese Medicine of South Hubei Province, Xianning, Hubei, China; ^3^ Faculty of Chinese Medicine and State Key Laboratory of Quality Research in Chinese Medicine, Macau University of Science and Technology, Macau, Macao SAR, China

**Keywords:** Maximum Entropy model, *Hovenia dulcis*, environmental variable, contribution rate, confidence importance, potential distribution area

## Abstract

**Introduction:**

*Hovenia dulcis* Thunberg, a multifunctional medicinal plant native to East and Southeast Asia, has been introduced worldwide. However, the environmental factors that determine its habitat and its precise distribution in China remain incompletely characterized.

**Methods:**

Therefore, the Maximum Entropy (MaxEnt) model integrated with, ArcGIS was employed to predict the potential distribution of *H. dulcis* in China, using 479 initial occurrence records (which were spatially filtered to 191 points) and 33 environmental variables (of which 15 were selected for the final analysis). Model performance was assessed via AUC-ROC, with key variables identified through permutation importance and response curves. Future projections were made under SSP126 and SSP585 scenarios for the 2050s and 2090s.

**Results:**

The model demonstrated high accuracy (AUC = 0.934). The distribution of *H. dulcis* was primarily governed by annual precipitation (Bio12), the minimum temperature of the coldest month (Bio06), elevation, and the mean diurnal temperature range (Bio02). The optimal ranges for these variables were as follows: annual precipitation of 708.5–2,956.8 mm, a minimum temperature of the coldest month between -4.9 and 8.9 °C, elevation of 273.9–1,019.4 m, and a mean diurnal temperature range of 6.81–10.18 °C. At present, suitable habitats are concentrated in central and southwestern China. Future projections indicate a northward shift and altitudinal increase in suitable areas, with expansions in Beijing, Hebei, and Liaoning, but contractions in Guangxi and Shandong. Hunan, Jiangxi, Sichuan, and Guizhou remain core suitable regions. This northward shift is consistent with preference of *H. dulcis* for the warm temperatures and adequate humidity, highlighting both its vulnerability and its adaptive potential under global warming.

**Discussion:**

*H. dulcis* is highly sensitive to climatic variables, particularly temperature and precipitation. Our findings provide a scientific basis for developing well-targeted conservation strategies, promoting sustainable utilization, and optimizing cultivation practices for *H. dulcis* under climate change.

## 1 Introduction

*Hovenia dulcis* Thunberg, a member of the Rhamnaceae family, is a multifunctional plant with significant medicinal and economic value (De Godoi et al., 2021). It is native to East and Southeast Asia and has been introduced and naturalized in North America, Australia, and New Zealand (Sferrazza et al., 2021). It is worth noting that the species has fragrant flowers and possesses large leaves, which contributes to its considerable value in urban air regulation and make it a suitable candidate for street greening. Its fruits and seeds, known as “Zhijuzi” in traditional Chinese medicine, are well supported by evidence to have hepatoprotective effects (Hyun et al., 2010). For centuries, “Zhijuzi” has been used to treat health conditions such as fever, excessive thirst, alcohol poisoning, and urinary disorders. Pharmacological studies have confirmed its broad bioactive properties, especially its ability to reduce blood alcohol levels and enhance alcohol metabolism (Meng et al., 2020), while exhibiting significant antioxidant activity that mitigates alcohol-induced oxidative stress (Tomczyk et al., 2012). The phytochemical profile of *H. dulcis* is remarkably diverse, comprising constituents such as flavonoids, terpenoids, fatty acids, saponins, and polysaccharides, which exhibit potential therapeutic effects against a range of liver diseases, particularly those associated with alcohol consumption (Li et al., 2021; He et al., 2024).

Global warming profoundly affects ecosystems worldwide and threatens both the geographic distribution and long-term persistence of medicinal plant species. As climatic change increasingly accelerates in rate and magnitude, assessing its impacts has become an urgent matter that needs to be prioritized (Thomas et al., 2004). In China, changes in climatic conditions have intensified the occurrence of extreme meteorological events. The frequency and severity of such events are both on the rise, exerting a profound impact on the ecological environment and threatening the natural habitats of numerous plant species. Environmental variables, especially temperature, precipitation, and elevation, together with human activities, fundamentally shape the distribution and quality of medicinal plants. The prediction output of species distribution models under future climate scenarios largely depends on method selection (e.g., algorithm selection and predictor curation) and climatic uncertainties (e.g., divergent projection ranges and greenhouse gas trajectories) (Porfirio et al., 2014). Forecasting the potential geographical distribution of species under climate change conditions is essential for biodiversity conservation and the sustainable utilization of resources (Guisan and Thuiller, 2005).

Species distribution models (SDMs) offer critical insights into the exploration and prediction of species distribution and play an essential role in understanding and conserving global biodiversity (Niiya et al., 2024). Various SDMs are commonly used to assess potential species habitats, including Maximum Entropy (MaxEnt), Boosted Regression Trees (BRT), Random Forests (RF), Generalized Additive Model (GAMs), and Generalized Linear Model (GLMs) (Melo-Merino et al., 2020). According to statistical analyses, the MaxEnt model is the most widely used (Khan et al., 2022). Since its introduction in 2006, it has become a mainstream approach in species distribution modeling (Phillips et al., 2006). By applying the principle of maximum entropy, it effectively identifies the most influential environmental factors, even under complex ecological conditions (Cao et al., 2021). There are three main reasons why the MaxEnt model stands out. First of all, it is specifically designed for presence-only data, which is particularly valuable for medicinal plants with insufficient sampling. Secondly, it performs reliably with small sample sizes of as few as 25 records, while minimizing overfitting through built-in regularization (Phillips and Dudík, 2008). Finally, its logistic output generates continuous probability surfaces, facilitating the interpretation of protection planning. Therefore, MaxEnt has been widely applied in disciplines such as conservation biology and ecology (Kumar et al., 2022; Shen et al., 2023).

To examine the current and future distribution patterns of *H. dulcis* across China, this study integrates species occurrence records with climatic, soil and topographic variables. Using MaxEnt model in combination with ArcGIS spatial analysis, we reconstruct habitat suitability during the Last Glacial Maximum (LGM) and Mid-Holocene (MH) scenarios, evaluate current suitability from 1970 to 2000, and project future distributions for the 2050s and 2090s. The study aims to achieve four key objectives: (1) to simulate the current potential distribution and delineate suitable habitats of *H. dulcis*; (2) to identify the dominant ecological variables that control its geographical scope; (3) to forecast shifts in suitable habitats for the 2050s and 2090s through analyses of response curves and permutation importance; and (4) to elucidate optimal growth conditions, thereby providing a theoretical foundation for conservation and sustainable utilization strategies.

Additionally, these findings will support the development of strategies for the protection, cultivation, and sustainable utilization of *H. dulcis*, thereby ensuring its long-term survival and ecological contributions under global warming. By delineating optimal habitats and identifying the dominant environmental variables, the study elucidates both the most favorable growth conditions and the potential impacts of climate change (Li and Park, 2020). These insights will strengthen the conservation and management of *H. dulcis* resources, ensuring their continuous availability for both medicinal and economic applications.

## 2 Materials and methods

### 2.1 Acquisition and screening of *H. dulcis* distribution data

**Figure 1:**
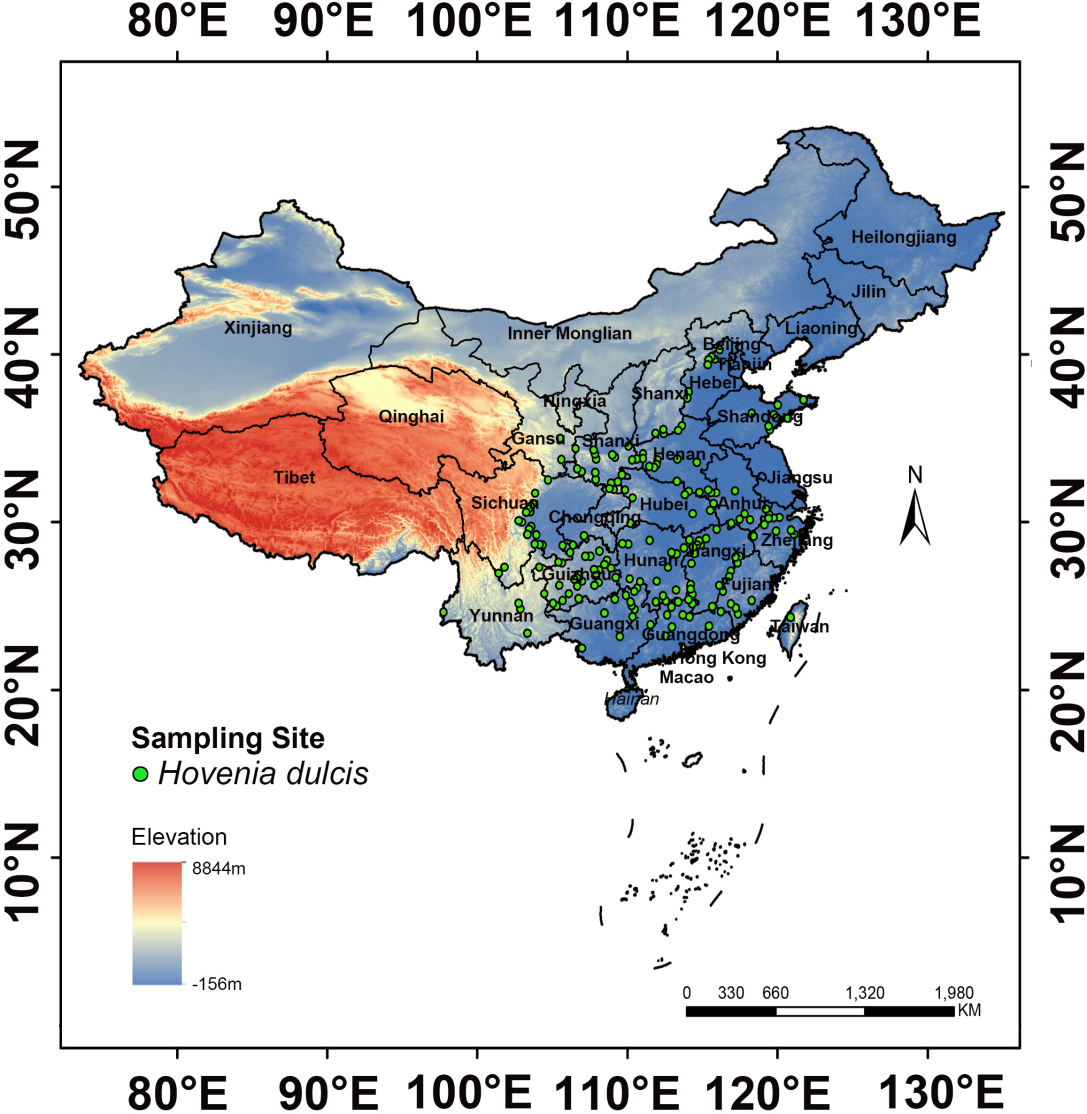
Distribution map of *H. dulcis*.

To systematically investigate the distribution of *H. dulcis*, occurrence data were retrieved from major online botanical databases, including the Chinese Virtual Herbarium (CVH, https://www.cvh.ac.cn/) and the China National Specimen Information Infrastructure (NSII, http://www.nsii.org.cn/). A total of 479 occurrence records across China were compiled (**Supplementary Table S1**). To remove duplicate and ambiguous entries, records lacking precise geographical coordinates were georeferenced using the Baidu Coordinate Pickup System (http://api.map.baidu.com/lbsapi/getpoint/index.html) (Fan et al., 2014). This process resulted in 254 accurately georeferenced occurrence points (**Supplementary Table S2**). To reduce overfitting caused by sampling bias, spatial filtering was performed in ArcGIS 10.4.1. A 10 km buffer was generated around each point, and within every 20 km diameter circle, a single presence record was randomly retained. The filtering procedure was determined primarily by three considerations: the ecological traits of *H. dulcis*, a deciduous tree with animal-assisted seed dispersal, which justify the use of a 10 km buffer to account for local clustering (Zhou et al., 2013); the spatial resolution of environmental variables, such as bioclimatic, soil and topographic variables exhibits spatial autocorrelation within 10 km, making a 20 km zone appropriate for capturing environmental variation (Pokharel et al., 2016); methodological consistency, as similar filtering thresholds of 10–20 km have been widely applied to medicinal plants such as *Panax notoginseng*, *Piper kadsura*, and *Magnolia biondii* to balance ecological accuracy with bias control (Guo et al., 2025; Li et al., 2025). This procedure yielded 191 validated occurrence points (**Figure 1**; **Supplementary Table S3**), which were formatted into a CSV file containing the species name, longitude, and latitude for subsequent modeling. According to the NSII platform and Flora of China, these records were primarily distributed across central, eastern and southwestern China. The highest numbers of records were from Henan (66 points), Jiangxi (64 points), Guizhou (40 points), Shandong (33 points), Shaanxi (28 points), Hebei (23 points), Sichuan (22 points), Hunan (20 points), Guangxi (19 points), Fujian and Zhejiang (17 points each), Anhui (16 points), and Hubei (13 points).

### 2.2 Acquisition and screening of environmental variables influencing suitable habitats

Nineteen bioclimatic variables were obtained from the WorldClim database (http://www.worldclim.org). The soil variables utilized in our study were collected from the World Soil Database hosted on the FAO Soils Portal (http://www.fao.org/soils-portal/data-hub/en/), totaling 11 distinct parameters. The topographic variables were sourced from the WorldClim website (https://www.worldclim.org/), amounting to 3 variables (Ouyang et al., 2022). Overall, these 33 independent environmental variables form a comprehensive dataset that has been carefully selected and prepared for the ecological analyses outlined in **Table 1**. We adopted the 1970–2000 climate dataset as our baseline, supplemented with historical climate data from LGM and MH scenarios, as well as future climate projections for 2041–2060 and 2081–2100 under different emission scenarios. For future climate projections, we used the CMIP6-based Shared Socioeconomic Pathways (SSPs) framework, which defines alternative socio-economic and climate change scenarios. Among them, SSP126 represents a low-emission scenario and SSP585 represents a high-emission scenario, both are widely used to predict climate change impacts on species distributions (Karuppaiah et al., 2023). These scenarios have been employed in numerous ecological predictions, including the research on medicinal plants such as *Zingiber striolatum* and various pests and plants (Huang et al., 2024; Mao et al., 2024). While intermediate scenarios aid in a more comprehensive understanding of impacts across different emission trajectories, many studies (including ours) have chosen extreme scenarios to simplify analysis and conserve computational resources (Zheng et al., 2022).

**Table 1:** Description of environmental variables.

Variable	Description	Variable	Description
Bio1	Annual average temperature (°C)	Bio18	Precipitation of warmest quarter (mm)
Bio2	Mean diurnal range (mean of monthly (max temp - min temp)) (°C)	Bio19	Precipitation of coldest quarter (mm)
Bio3	Isothermality (bio2/bio7) (× 100)	awc_class	Soil available water content
Bio4	Temperature seasonality (standard deviation × 100)	s_caco3	Topsoil calcium Carbonate (%wt)
Bio5	Max temperature of warmest month (°C)	s_clay	Substrate-soil clay content (%wt)
Bio6	Min temperature of coldest month (°C)	s_oc	Substrate-soil organic carbon (%wt)
Bio7	Annual temperature span (bio5-bio6) (°C)	s_ph_h2o	Substrate-soil pH
Bio8	Mean temperature of wettest quarter (°C)	s_sand	Sediment content in the subsoil (%wt)
Bio9	Mean temperature of driest quarter (°C)	t_caco3	Topsoil carbonate or lime content (%wt)
Bio10	Mean temperature of warmest quarter (°C)	t_clay	Clay content in the upper soil (%wt)
Bio11	Mean temperature of coldest quarter (°C)	t_oc	Topsoil organic carbon (%wt)
Bio12	Annual precipitation (mm)	t_ph_h2o	Topsoil pH
Bio13	Precipitation of wettest month (mm)	t_sand	Sand content (%wt)
Bio14	Precipitation of driest month (mm)	aspect	Aspect
Bio15	Precipitation variability (coefficient of variation)	elev	Elevation (m)
Bio16	Rainfall of wettest quarter (mm)	slope	Slope (°)
Bio17	Precipitation of driest quarter (mm)		

### 2.3 Correlation analysis and determination of key environmental variables for adaptation

To mitigate multicollinearity among environmental variables and reduce the risk of overfitting, we calculated Spearman’s rank correlations in SPSS 26.0. We excluded any variable that exhibited |r| > 0.8 with another variable and whose permutation-based variable importance score contributed < 5% to the ensemble model. According to the describe screening procedure (Zhang et al., 2020), 15 variables for the *H. dulcis* distribution model were finally selected. It included 7 bioclimatic variables (Bio_2, Bio_3, Bio_4, Bio_6, Bio_8, Bio_10, and Bio_12), 5 edaphic variables (top-soil organic carbon, sub-soil organic carbon, calcium carbonate content, sand, and clay fractions), and 3 topographic variables (elevation, slope, and aspect).

### 2.4 Construction of the MaxEnt model

Climate variables and species-occurrence records of *H. dulcis* were inputted into MaxEnt, with the parameters set as: bootstrap resampling, logistic output, and the default regularization multiplier of 1. This follows common practices in similar studies when lacking species-specific tuning data, and preliminary tests showed no significant performance improvement with adjustments (Zhan et al., 2022). Although the logistic output was optional in MaxEnt, it yielded an estimate of occurrence probability that is more readily interpretable (Elith et al., 2011). And 75% of the occurrence records were randomly selected as the training set, and the remaining 25% were used as the test set to evaluate the model performance. Each bootstrap replicate was run for 1000 iterations, which was consistent with the default setting commonly used in species distribution modeling studies (Pischl et al., 2020), and the ensemble average of 10 replicates was adopted as the final prediction (Syfert et al., 2013).

Raster outputs for *H. dulcis* were imported into ArcMap 10.4.1 and reclassified using the natural-breaks method (Bergamin et al., 2022). Subsequently, the area under the receiver operating characteristic curve (AUC-ROC) was used to evaluate the validity of the model. As a threshold-independent metric, AUC-ROC has been emphasized in recent studies for evaluating the MaxEnt model (Ahmadi et al., 2023). The predictive accuracy of model was classified according to standard AUC thresholds, <0.6 indicated failure, 0.6–0.7 represented poor, 0.7–0.8 showed moderate, 0.8–0.9 demonstrated good, and 0.9–1.0 means excellent predictive performance (Ottó and Végvári, 2022).

### 2.5 Model evaluation and habitat classification

The Jackknife method was used to evaluate the relative influence of individual environmental variables on the distribution of *H. dulcis* (Hong et al., 2021). Response curves of the most influential variables were generated to visualize the environmental preferences of species (Yan et al., 2020; Son et al., 2023). Ranking importance quantified model sensitivity to each variable by randomly changing its values across training and background data, with higher values indicating greater influence (Ma et al., 2024).

In species distribution modeling and habitat classification, the Maximum Test Sensitivity plus Specificity (MTSPS) threshold was widely applied to distinguish suitable from unsuitable habitats because of its practicality and effectiveness. The mean of the ten MTSPS values was then adopted as the determination threshold, and habitat suitability was classified into four categories: unsuitable (0–MTSPS), low suitability (MTSPS–0.3), medium suitability (0.3–0.5), and high suitability (0.5–1) (Aligaz et al., 2024). This procedure ensured that the threshold is derived exclusively from data not used in training, minimizing overfitting and ensuring the objectivity and generalizability of the classification criterion, in line with best-practice recommendations in species-distribution modeling. The occurrence probability of *H. dulcis* was projected throughout China. Furthermore, the suitability models were applied to seven different scenarios to generate corresponding distribution maps, including the Last Glacial Maximum (LGM), Mid-Holocene (MH), current, and future projections for the 2050s and 2090s under both SSP126 and SSP585. Based on the MTSPS classification standard, the areas of medium- and high-suitability habitats were calculated, and their sum was determined as the total suitable habitat area (Zhang et al., 2023; Yang et al., 2024).

### 2.6 Analysis of the area changes of the suitable habitat of different provinces in China

The provincial boundary shapefile of China (Review Map No.: GS(2019)1822) was imported into ArcMap 10.4.1. Habitat suitability rasters (.asc) for each period were converted to GeoTIFF (.tif) format with FLOAT data type. After assigning the WGS_1984 geographical coordinate system, the data were projected to WGS_1984_Albers for accurate area measurement. Provincial attribute tables were updated with the corresponding province names. Then, the continuous suitability data were reclassified into four categories: unsuitable, low suitability, medium suitability, and high suitability. The regional geometry tool calculated categorical areas within each province. The results were exported as dBASE files for quantitative analysis in Excel. Under different scenarios, the growth rate of suitable habitat area for each province was calculated as the percentage increase relative to the current suitable habitat area. Venn diagrams were generated using Microbioinformatics (http://www.bioinformatics.com.cn/) to visualize provincial habitat distribution patterns (Tang et al., 2023).

### 2.7 Statistical correlation with climatic variables

To explore the potential drivers of observed habitat changes, provincial-level meteorological data for 2021, 2022, and 2024 (2023 data were unavailable) were obtained from the National Meteorological Science Data Center (https://data.cma.cn/; **Supplementary Table S6**). Spearman’s rank correlation analysis was performed in Origin to quantify the relationships between provincial rates of habitat expansion or contraction and key climatic variables. The statistical significance of the correlations was evaluated using a p-value threshold of 0.05. The results were visualized as correlation heatmaps.

## 3 Results

### 3.1 Model accuracy analysis

The potential distribution of *H. dulcis* was predicted using the MaxEnt model. The model was run for 10 replicates, and the results were combined into an ensemble average. Model performance was assessed using the area under the receiver operating characteristic curve (AUC). To address potential overfitting issue that may caused by multicollinearity, we performed a Spearman’s rank correlation analysis on all 33 environmental variables.

**Figure 2:**
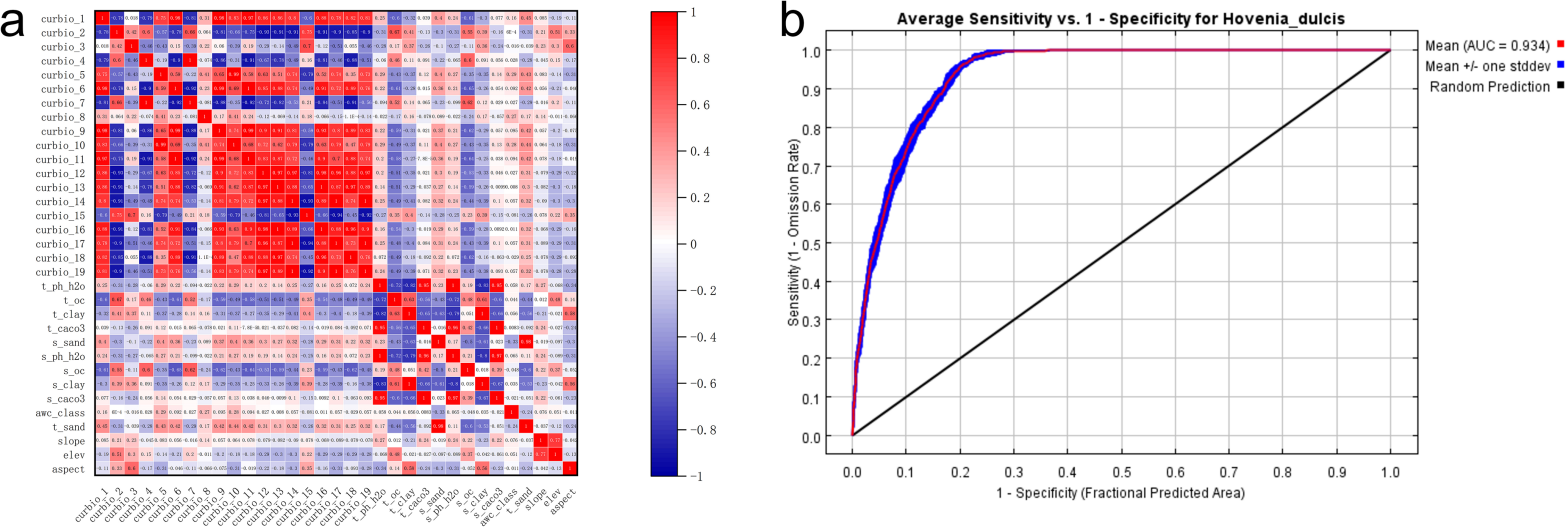
Prediction of the suitable habitat of *H*. *dulcis* based on the MaxEnt model and analysis of environmental variables. **(a)** Correlation heatmap related to environmental variables of *H. dulcis*; **(b)** ROC curve of the MaxEnt model.

In this study, the average training AUC for *H. dulcis* was 0.934 (**Figure 2b**), while the test AUC based on an independent subset of 25% of occurrence records was 0.921. Both values exceeded 0.9, indicating excellent predictive performance and strong model reliability. The approach applied in this study effectively identified key environmental variables shaping species distribution, which was in line with established methods of ecological modeling.

### 3.2 Identification of key environmental variables

Using the MaxEnt algorithm, the relative contributions of 15 environmental variables to the species distribution model were evaluated based on percentage contribution and permutation importance. In **Table 2**, Annual precipitation (Bio12) was the most influential factor (39.5%), followed by the minimum temperature of the coldest month (Bio06, 31.1%). Additional variables with measurable effects included slope (9.4%) and isothermality (Bio03, 5.8%), while the contributions of all remaining variables were minor (≤2.8%). Through the assessment of permutation importance, reflecting the sensitivity of the model, the primary influence of Bio06 (31.2%), elevation (16.9%), and Bio12 (16.6%) were confirmed, highlighting their critical roles in shaping the distribution of *H. dulcis*.

**Table 2:** Percent contribution and permutation importance of the dominant environmental variables in the MaxEnt model.

Variable	Description	Percent contribution (%)	Permutation importance (%)
bio12	Annual precipitation	39.5	16.6
bio06	Min temperature of coldest month	31.1	31.2
slope	Slope	9.4	10.3
bio03	Isothermality ((Bio02/Bio07) * 100)	5.8	3.5
elev	Elevation	2.8	16.9
aspect	Aspect	2.5	2.2
bio02	Mean diurnal range (mean of monthly (max temp - min temp))	2.0	3.0
bio04	Temperature seasonality	1.5	4.5
s_clay	Substrate-soil clay content	1.5	2
s_caco3	Topsoil calcium Carbonate	1.3	2.7
bio08	Mean temperature of wettest quarter	1.1	1.4
s_sand	Sediment content in the subsoil	0.8	3.7
s_oc	Substrate-soil organic carbon	0.5	0.5
t_oc	Topsoil organic carbon	0.3	0.4
bio10	Mean temperature of warmest quarter	0.2	0.8

**Figure 3:**
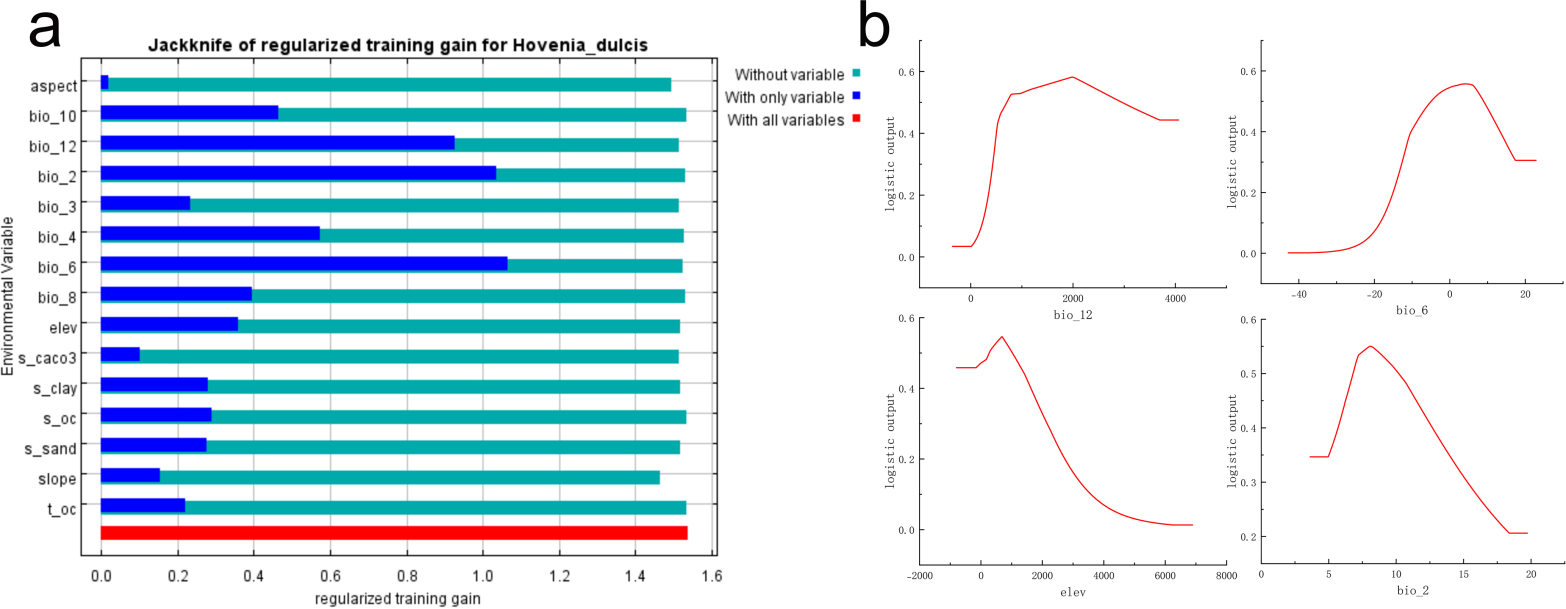
Prediction of the suitable habitat of *H*. *dulcis* based on the MaxEnt model. **(a)** Jackknife test of environmental variables; **(b)** Response curve of key influential variables.

The Jackknife test further underscored the critical importance of these variables for mapping suitable habitats for *H. dulcis* across China. Specifically, Bio06 (Minimum Temperature of the Coldest Month), Bio02 (Mean Diurnal Range), and Bio12 (Annual Precipitation) emerged as the most influential variables governing its distribution (**Figure 3a**). Therefore, the distribution of the species was mainly driven by extreme temperatures, diurnal temperature variation, annual precipitation, and topography (elevation).

Response curve analysis (**Figure 3b**) identified the optimal ranges and threshold values of the key environmental variables that restrict the distribution of *H. dulcis*. The appropriate ranges and corresponding optimum values were: annual precipitation of 708.45–2956.80 mm (Bio12; optimum: 1985.02 mm), minimum temperature of the coldest month from –4.93 to 8.92°C (Bio06; optimum: 4.20°C), elevation between 273.85 and 1019.40 m (optimum: 681.21 m), and mean diurnal temperature range of 6.81–10.18 °C (Bio02; optimum: 8.13°C). Within these intervals, the probability of species occurrence increased toward the optimum value, whereas values beyond these thresholds resulted in a reduced probability of occurrence. Overall, temperature-related variables, precipitation, and elevation were the primary environmental driving factors affecting the distribution of *H. dulcis.*

### 3.3 Distribution prediction of *H. dulcis* under current climate conditions

**Figure 4:**
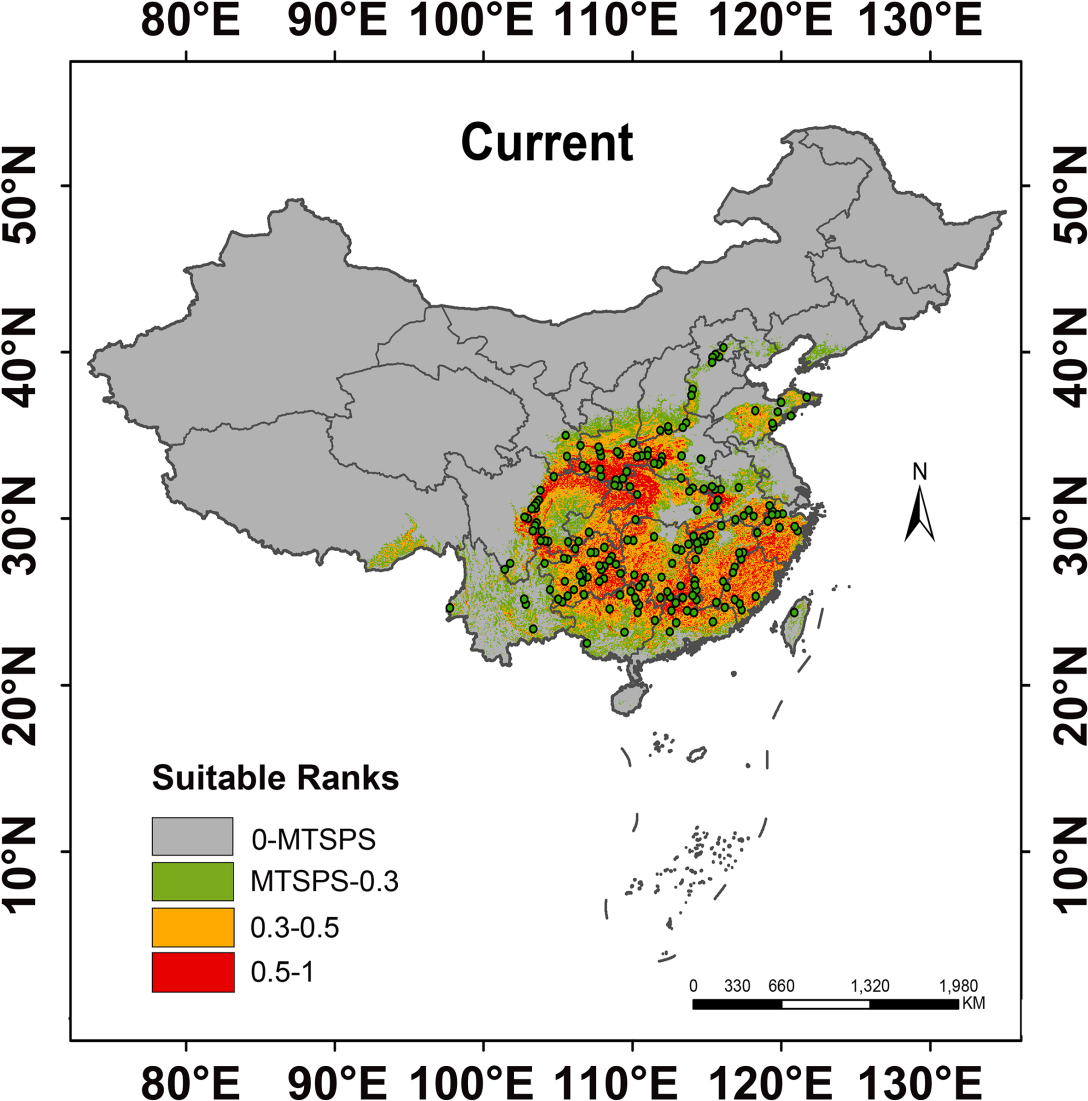
Distribution of suitable habitats for *H. dulcis* in the current scenario.

The predicted distribution of suitable habitats for *H. dulcis* under current climate conditions was visually summarized in **Figure 4**. Habitat suitability was classified into four categories: unsuitable (gray), low-suitable (green), medium-suitable (yellow), and high-suitable (red). It is primarily distributed between 30°N–37°N latitude and 101°E–123°E longitude, delineating its overall suitable habitat range. The total suitable area was estimated at 147.70 × 10^4^ km², accounting for 15.39% of China’s land area, among which high-suitable habitats accounted for 35.23%. The total suitable habitat area of *H. dulcis* was relatively concentrated, primarily located at the intersection of central, southwestern, and northwestern China, as well as coastal regions of eastern and southern China. No suitable habitats were predicted in the northernmost parts of the country. Occurrence records further indicate that *H. dulcis* predominantly occupies low- to mid-elevation hilly terrain, particularly around the Sichuan Basin. This predicted distribution was consistent with the range of the species’ native habitats recorded in the *Flora of China*, and also closely corresponded to the specimen records of the herbarium from 1950 to 2020. By contrast, unsuitable habitats were mostly located in northeastern, northern, and northwestern China, which might be due to the limitations of climatic factors.

### 3.4 Distribution prediction under past and future climates

**Figure 5:**
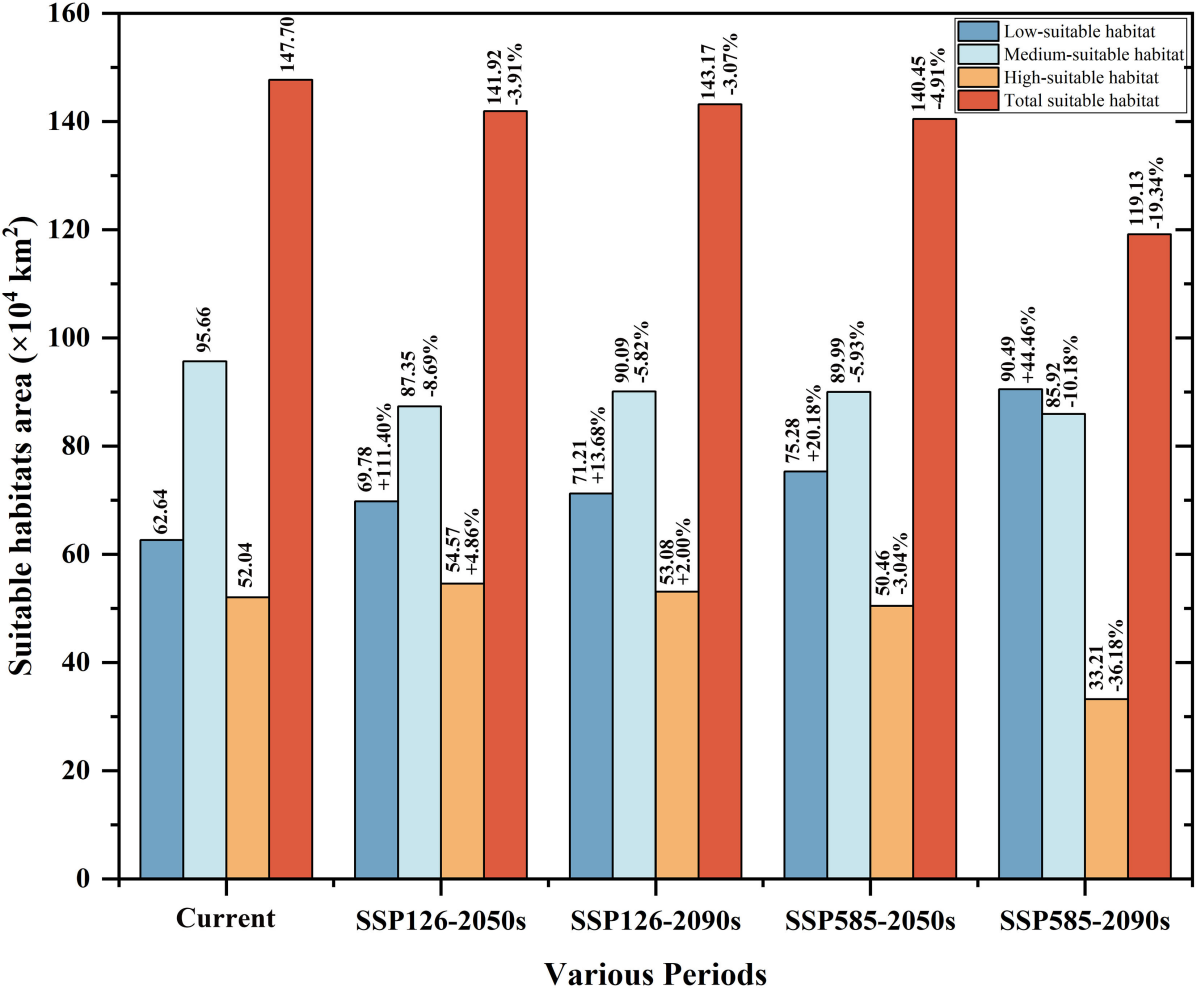
Suitable habitat area of *H. dulcis* under future climate conditions and the change in area compared to the current climate.

**Figure 6:**
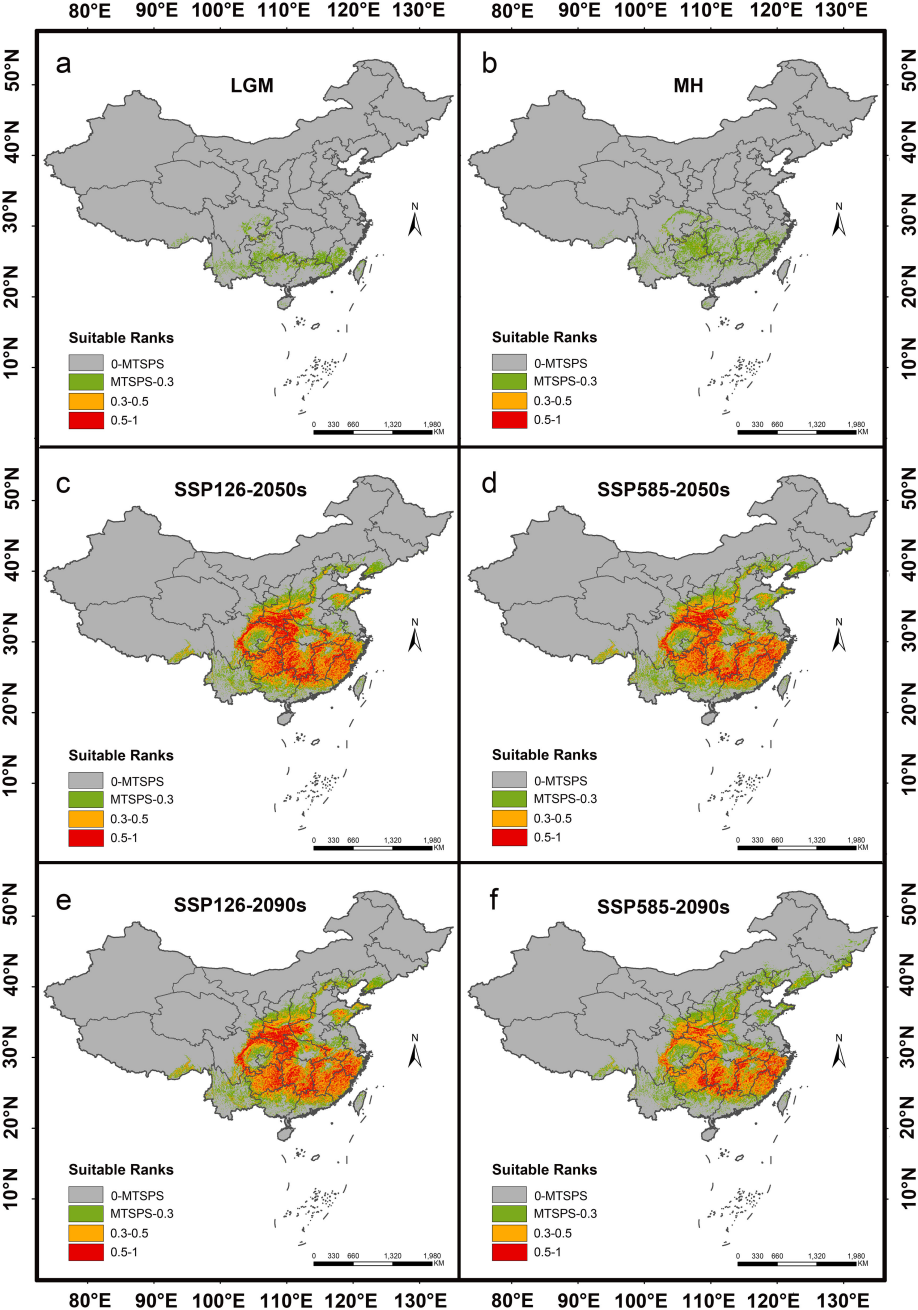
Distribution of suitable habitats for *H*. *dulcis* under different climate scenarios. **(a)** Last Glacial Maximum (LGM); **(b)** Mid-Holocene (MH); **(c)** 2041–2060 (2050s) average, SSP126; **(d)** 2041–2060 (2050s) average, SSP585; **(e)** 2081–2100 (2090s) average, SSP126; **(f)** 2081–2100 (2090s) average, SSP585.

The past and future predicted distribution patterns of *H. dulcis* were summarized in **Table 3** and **Figures 5**, **6**. Under the LGM and MH scenarios, the total area of suitable habitat was markedly restricted, with most habitats classified as low suitability (**Figures 6a, b**). Although the MH scenario showed a wider range of suitable environments than the LGM scenario, no high-suitable habitats were detected, and medium-suitable habitats remained limited. This pattern was consistent with the key climatic variables identified to affect the distribution of *H. dulcis*, especially the minimum temperature of the coldest month and annual precipitation.

**Table 3:** Statistics on the area of the suitable habitat of *H. dulcis* in different periods.

Period		Unsuitable habitat		Low-suitable habitat		Medium-suitable habitat		High-suitable habitat	
		Area (*10^4^ km^2^)	Percentage (%)	Area (*10^4^ km^2^)	Percentage (%)	Area (*10^4^ km^2^)	Percentage (%)	Area (*10^4^ km^2^)	Percentage (%)
LGM		927.45	96.61	31.04	3.23	1.51	0.16	0	0
MH		913.91	95.20	44.39	4.62	1.70	0.18	0	0
Current		749.66	78.09	62.64	6.53	95.66	9.96	52.04	5.42
2050s	SSP126	748.30	77.95	69.78	7.27	87.35	9.10	54.57	5.68
	SSP585	744.27	77.53	75.28	7.84	89.99	9.37	50.46	5.26
2090s	SSP126	745.62	77.67	71.21	7.42	90.09	9.38	53.08	5.53
	SSP585	750.38	78.16	90.49	9.43	85.92	8.95	33.21	3.46

At present, the suitable habitat area of *H. dulcis* has expanded considerably. High-, medium-, and low-suitable habitats covered 52.04 × 10^4^ km², 95.66 × 10^4^ km², and 62.64 × 10^4^ km² respectively (**Figure 5**). These findings indicated that contemporary climatic conditions are favorable for its survival. The core distribution area was in subtropical monsoon zones, with hot and humid summers and mild and moist winters. Such climatic conditions were consistent with the key environmental variables identified in the response analysis.

Future projections indicated a general contraction of suitable habitats (**Figures 6c-f**). Under the SSP126 scenario, it was expected that the appropriate area will initially decrease and then rebound slightly, but it would still be lower than the current levels. Between 2041 and 2060, the total suitable area was expected to decline by 3.91% to 141.92 × 10^4^ km^2^. Medium-suitable habitats were projected to decrease by 8.69%, whereas low- and high-suitable habitats were expected to increase by 11.40% and 4.86% respectively.

From 2081 to 2100, the estimated total suitable area was 143.17 × 10^4^ km^2^, a decrease of 3.07% compared to current conditions. During this period, low- and high-suitable habitats were expected to increase by 13.68% and 2.00% respectively, whereas medium-suitable habitats were projected to decline by 5.82%. However, under the SSP585 scenario, the loss of suitable habitat was predicted to be more pronounced. Between 2041 and 2060, the total suitable area was projected to decline by 4.91% to 140.45 × 10^4^ km^2^. Low-suitable habitats were expected to increase by 20.18%, while medium- and high-suitable habitats decreased by 5.93% and 3.04% respectively. It was estimated that from 2081 to 2100, the total suitable area will decline sharply by 19.35% to 119.12 × 10^4^ km^2^. Among them, the low-suitable habitats increased by 44.46%, medium-suitable habitats decreased by 10.18%, and high-suitable habitats declined by 36.18%. Collectively, these projections indicated that global warming will substantially reduce the environmental suitability for *H. dulcis* survival.

### 3.5 Provincial distribution of suitable habitats for *H. dulcis* under current climate condition

Under current climatic conditions, the distribution of suitable habitats for *H. dulcis* varied considerably across Chinese provinces and was broadly spread across many regions (**Supplementary Table S4**). No suitable habitats were found in Heilongjiang, Shanghai, Xinjiang, or Macau SAR, while other provinces had suitable habitats to varying degrees. Medium-suitable habitats were concentrated in central and western China, while high-suitable habitats were distributed across western, central, and eastern regions. As shown in **Table 1**, Yunnan Province had the largest area of low-suitable habitat, covering 199,295.91 km^2^. The Guangxi Zhuang Autonomous Region contained the largest extent of medium-suitable habitat (95,434.07 km^2^), followed by Hunan (95,047.70 km^2^), Sichuan (82,345.70 km^2^), Guizhou (79,979.16 km^2^), and Jiangxi (73,048.60 km^2^). All other provinces contained less than 60,000 km² of medium-suitable habitat.

Hunan Province contained the largest area of high-suitable habitat, covering 85,364.23 km^2^, followed by Guizhou (79,544.49 km^2^), Sichuan (75,656.62 km^2^), Hubei (74,714.83 km^2^), Jiangxi (70,778.66 km^2^), Shaanxi (65,079.66 km^2^), and Fujian (62,495.80 km^2^). Each of the remaining provinces has less than 60,000 km^2^ of high-suitable habitat. Under the current climatic conditions, the combination of medium- and high-suitable habitats indicated that Hunan, Guizhou, Sichuan, and Jiangxi are the most suitable regions for the growth of *H. dulcis*.

### 3.6 Changes in the suitable habitat of *H. dulcis* under SSP126 and SSP585 scenarios

Further analysis of **Figure 6** and **Supplementary Table S5** illustrated changes in suitable habitats of *H. dulcis* under two future climate scenarios. Under the SSP126 scenario, suitable habitats across China were projected to expand in both the 2050s and the 2090s. Hebei, Liaoning, Ningxia, and Beijing exhibited continuous and significant expansion during both periods, with Hebei showing the greatest increase (216.93%) by the 2090s. In the 2050s, Shanxi and Gansu displayed the highest growth rates (70.03% and 33.67%, respectively), whereas Guangxi and Guangdong experienced declines of 23.40% and 17.70%. Among provinces with suitable habitat areas exceeding 15,000 km^2^, more exhibited habitat loss than expansion, while those below this threshold showed more variable patterns. By the 2090s, the reductions in Guangxi and Guangdong intensified to 30.53% and 21.18% respectively. Chongqing shifted from habitat reduction in the 2050s to expansion in the 2090s, while Yunnan followed the opposite trend. Overall, the number of provinces experiencing habitat loss exceeded those with gains, and the spatial pattern of habitat expansion and contraction remained largely consistent throughout the two periods.

Under the SSP585 scenario, Liaoning Province exhibited an exceptionally high growth rate of 1,936.36% by the 2090s, mainly due to the small baseline habitat area under current conditions. Therefore, even a moderate absolute increase resulted in a disproportionately high relative growth rate. Hebei, Shanxi, and Gansu also witnessed substantial increases, reaching 284.98%, 147.01%, and 121.36%, respectively, whereas Shandong and Guangxi saw an accelerated declines of 58.79% and 51.13%. Guizhou and Yunnan displayed more dynamic trends, with initial increases in the 2050s followed by declines in the 2090s. Among provinces with suitable areas exceeding 15,000 km^2^, the numbers of those gaining and losing habitats were roughly comparable, yet a net contraction was observed at the national scale. Both scenarios consistently revealed obvious north-south differences, with an increase in suitability in northern China and a decrease in suitability in southern regions.

**Figure 7:**
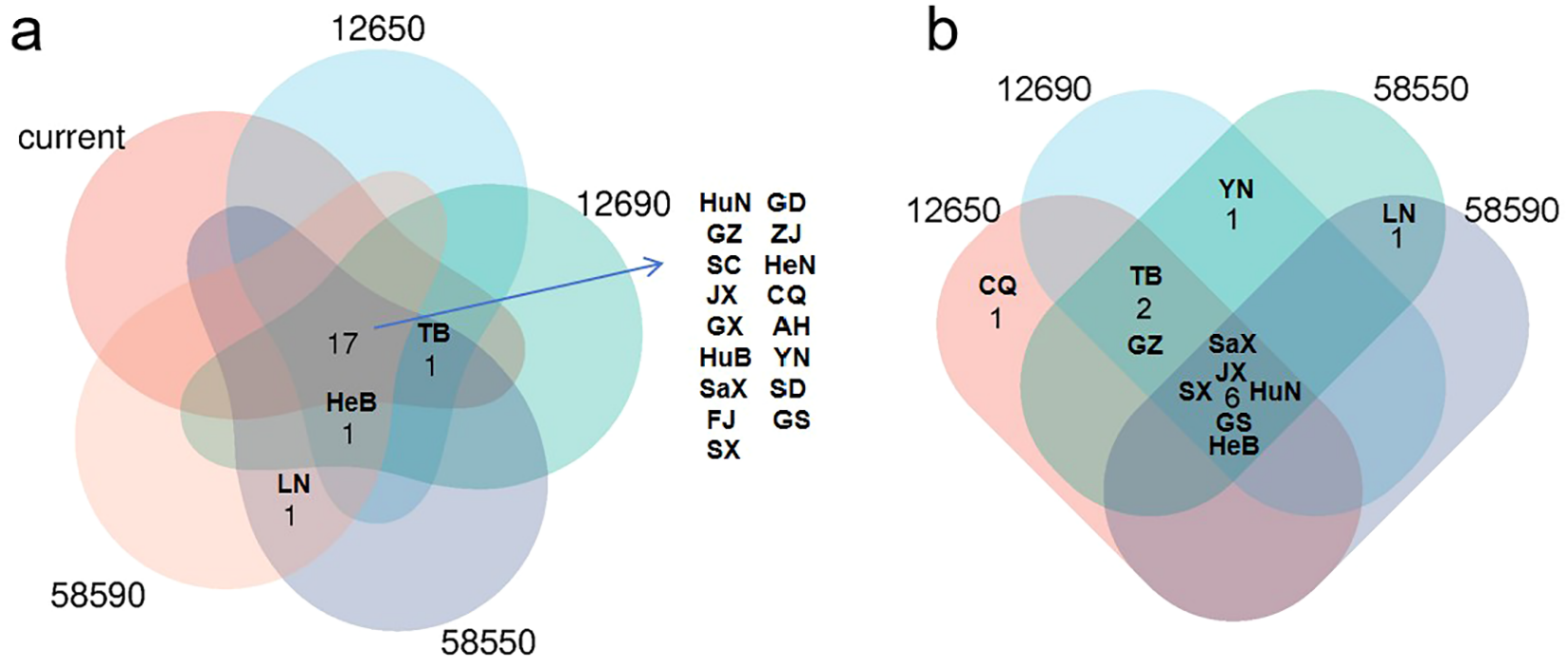
Provincial suitable habitat situation of *H*. *dulcis* under climate change scenarios. **(a)** Provinces where the area of suitable habitat remains above 15,000 km² throughout all periods and those expected to surpass 15,000 km^2^ in future periods; **(b)** Provinces where suitable habitat area consistently surpasses current climatic suitable habitat area. (HuN, Hunan Province; GZ, Guizhou Province; SC, Sichuan Province; JX, Jiangxi Province; GX, Guangxi Zhuang Autonomous Region; HuB, Hubei Province; SaX, Shaanxi Province; FJ, Fujian Province; SX, Shanxi Province; GD, Guangdong Province; ZJ, Zhejiang Province; HeN, Henan Province; CQ, Chongqing; AH, Anhui Province; YN, Yunnan Province; SD, Shandong Province; GS, Gansu Province; HeB, Hebei Province; TB, Tibet Autonomous Region; LN, Liaoning Province).

**Figure 8:**
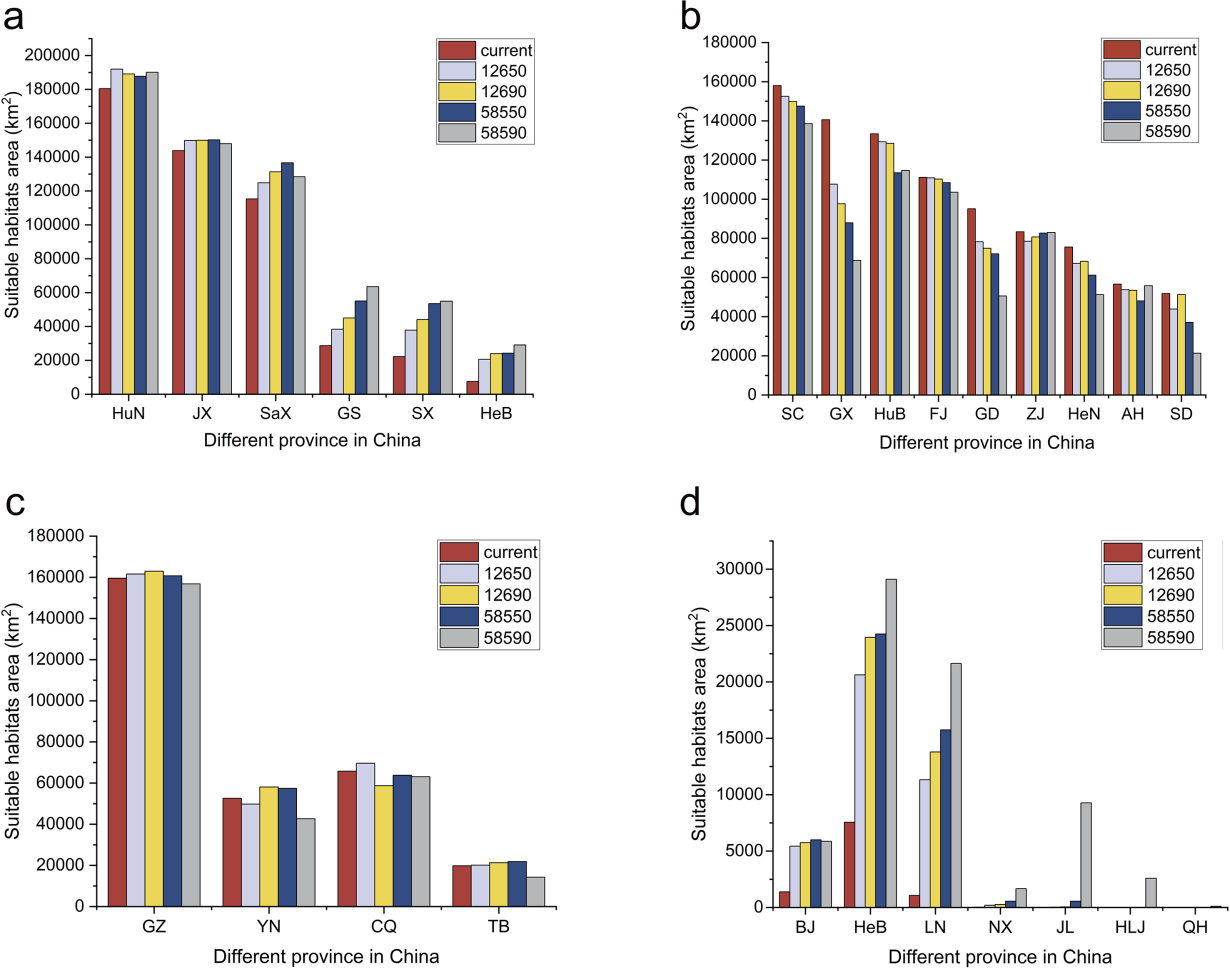
Area of suitable habitat in different provinces at different future climate scenarios. **(a)** The provinces with consistently larger suitable habitat areas than those under current climatic conditions; **(b)** Provinces with consistently smaller suitable habitat areas than under current climatic conditions; **(c)** Provinces where suitable habitat areas transiently exceed but ultimately remain smaller than current levels under future climate scenarios; **(d)** Provinces currently unsuitable but projected to become climatically suitable for *H*. *dulcis* under future climate scenarios.

According to statistical analysis, in both current and future scenarios, the suitable habitat in 17 provinces exceeded 15,000 km^2^ (**Figure 7a**). Among them, it was expected under future climate conditions, the temperatures in six provinces including Hebei, Shanxi, Jiangxi, Hunan, Shaanxi, and Gansu will continue to exceed current habitat levels (**Figures 7b**, **8a**). In contrast, Zhejiang, Anhui, Fujian, Shandong, Henan, Hubei, Guangdong, Guangxi, and Sichuan were expected to experience reductions in habitat suitability relative to current levels (**Figure 8b**). Guizhou, Yunnan, Chongqing, and Tibet maintained relatively stable suitable habitat areas, with only minor fluctuations under both present and future conditions (**Figure 8c**). It was worth noting that Beijing, Hebei, Liaoning, and Ningxia are expected to see a significant increase, while Jilin, Heilongjiang, and Qinghai are expected to see new suitable habitats, indicating a northward and mid-latitude shift in the potential distribution of *H. dulcis* (**Figure 8d**). In all scenarios, Hunan remained the most suitable province, while Jiangxi also sustained a large and continuously expanding area of suitable habitats. Although Guizhou and Sichuan were projected to lose some suitable area, they still maintained a relatively high degree of suitability.

### 3.7 Relationship between provincial habitat change and climatic variables

**Figure 9:**
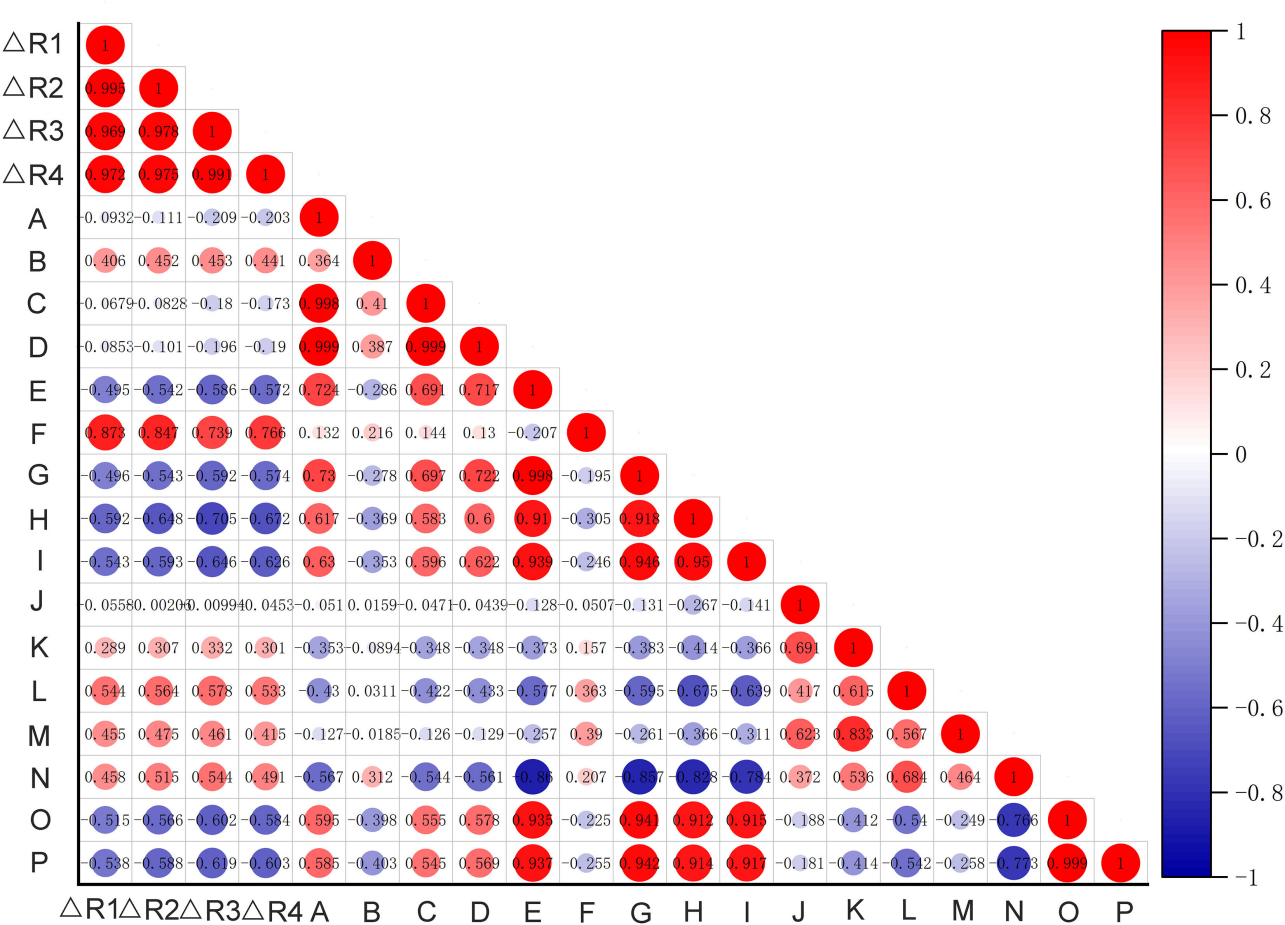
The correlation heatmap between the change rate of suitable habitat area in each province and climatic factors. (Hunan, Jiangxi, Shaanxi, Gansu, Shanxi, Hebei, Sichuan, Guangxi Zhuang Autonomous Region, Hubei, Fujian, Guangdong, Zhejiang, Henan, Anhui, Shandong)[△R1:△12650S/current; △R2:△12690S/current; △R3:△58550S/current; △R4:△58590S/current; (A) Atmospheric pressure (hPa); (B) Atmospheric pressure at sea level (hPa); (C) Maximum Pressure(hPa); (D) Minimum Pressure (hPa); (E) Temperature (°C); (F) Mean maximum temperature (°C); (G) Mean minimum temperature (°C); (H) Relative humidity (%); (I) Minimum Relative Humidity (%); (J) 2-Minute Mean Wind Speed (m/s); (K) Maximum Wind Speed (m/s); (L) Wind Direction (Angle) of Maximum Wind Speed (°); (M) Extreme Wind Speed (m/s); (N) Wind Direction of Extreme Wind Speed (°); (O) Annual 24-hour Precipitation (20:00-20:00 local time) (mm); (P) 24-hour Precipitation (08-08h) (mm)].

Analysis of meteorological conditions across provinces revealed that the projected change (future/current) in suitable habitat area for *H. dulcis* correlated positively with temperature, which was consistent with the thermophilic nature of this species (**Figure 9**). In contrast, the change rate showed significant and negative correlations with both precipitation and air humidity. This suggested that excessive moisture inhibits its growth, which might explain its absence in coastal regions. A weak positive correlation with wind speed implied that moderate winds may enhance gas exchange and stimulate physiological activity. Elevation exerted an indirect influence on distribution by interacting with topography and wind speed. Terrain features such as slopes and valleys altered local wind patterns, thereby modifying microclimatic conditions (such as temperature and moisture retention), which are crucial for *H. dulcis* survival. These findings aligned with prior analysis of key environmental variables, reaffirming the critical roles of temperature, precipitation, and elevation in determining habitat suitability. Consequently, our results supported the prediction of an overall range contraction for *H. dulcis* under future climate warming scenarios.

In summary, the priority provinces for future cultivation of *H. dulcis* included Hunan, Jiangxi, Hebei, Liaoning and Beijing, where the suitable habitat areas continued to expand. Meanwhile, attention should be paid to provinces such as Guizhou and Sichuan, where the suitable area was decreasing but still maintained high suitability.

## 4 Discussion

### 4.1 Reliability of MaxEnt model prediction

Species distribution models serve as the significant tool for assessing the impacts of climatic and environmental changes on habitat suitability (Wiens et al., 2009). Among them, MaxEnt model is regarded as one of the most frequently used ecological niche models in current studies, especially for presence-only data (Phillips and Dudík, 2008). This makes *H. dulcis* highly suitable for study, as a medicinal plant that has not received sufficient attention in biodiversity surveys. Our dataset contains 191 verified occurrence records. Although these records are spatially sparse, the robustness of the MaxEnt model effectively mitigates this limitation.

This study takes climatic conditions, edaphic properties, and topographical features as the main environmental variables and uses spatial modeling methods to predict the optimal habitat range of *H. dulcis*. However, the accuracy of the model predictions is influenced by the degree of spatial aggregation of occurrence records. When these records display a high level of spatial correlation, the model is prone to overfitting, potentially introducing geographical biases. To reduce overfitting, variables with correlation coefficients exceeding an absolute value of 0.8 are excluded. A 10 km spatial thinning threshold is applied to improve the accuracy and reliability of the AUC output. Model performance is evaluated using the receiver operating characteristic (ROC) curve, and the MaxEnt model achieves an AUC value of 0.934 (**Figure 2b**). An AUC value approaching 1 indicates excellent model performance (Mahmoud et al., 2025), demonstrating that the model is both accurate and effective. Its strong predictive capability provides a valuable reference for developing conservation strategies and sustainable utilization plans for *H. dulcis.*

### 4.2 Environmental variables influencing the distribution of *H. dulcis* and corresponding planting strategies

The distribution of *H. dulcis* is highly sensitive to climate and constrained by multiple environmental factors. Studies indicate that its potential distribution is primarily driven by four key variables: annual precipitation, minimum temperature of the coldest month, elevation, and mean diurnal temperature range. The species thrives under conditions of annual precipitation ranging from 708.45 to 2956.80 mm, minimum coldest-month temperatures between -4.93 °C and 8.92 °C, mean diurnal temperature ranges of 6.81–10.18 °C, and elevations from 273.85 to 1019.40 m. Optimal growth occurs at a minimum coldest-month temperature of 4.20 °C and a mean diurnal range of 8.13 °C, suggesting that *H. dulcis* prefers environments with relatively limited temperature fluctuations. The probability of occurrence shows a unimodal response to annual mean temperature (Bio1), with peaks ranging from 5.80 to 11.33 °C (Rong et al., 2024). Elevation strongly modulates regional climate and hence shapes plant distributions (Zhang et al., 2024).

In Northeast and North China, seasonal temperature variations are significant and no suitable habitats have been found, confirming that extreme temperature fluctuations limit survival. *H. dulcis* prefers warm and humid climates, with optimal growth occurring at an annual precipitation of 1985.02 mm, consistent with humid regions where precipitation typically exceeds 800 mm. Its distribution is concentrated in subtropical monsoon climate zones, primarily at the junction of Central, Southwest, and Northwest China, as well as in the coastal areas of East and South China. These regions are characterized by warm, moderately moist conditions and abundant rainfall, aligning closely with the model predictions and underscoring the dominant role of temperature and precipitation in shaping its distribution. Additionally, *H. dulcis* exhibits a strong preference for low- to mid-elevation hills ranging from 273.85 to 1019.40 m (Duan et al., 2025), consistent with its observed distribution (**Figure 1**). Therefore, conservation and cultivation efforts should give priority to warm, humid climates and mid-elevation areas to promote sustainable utilization.

### 4.3 Historical and future distribution evolution of the suitable habitat for *H. dulci*s under climate change

With global warming intensifying, the global surface temperature rose by 1.1°C during 2011–2020 compared with the baseline from 1850 to 1900, triggering significant redistributions and altered phenological timings that cascade into ecosystem-level reorganizations (Riahi et al., 2017). The frigid and arid conditions of the Last Glacial Maximum (LGM) are likely to have made the environment unsuitable for *H. dulcis* to survive. In contrast, the comparatively milder and wetter climate of the Mid-Holocene (MH) provided more favorable conditions for *H. dulcis.* (Berman et al., 2018). As a thermophilic and hydrophilic pioneer species, *H. dulcis* may have limited suitable habitats during both the LGM and MH scenarios, primarily constrained by extreme cold and unstable climate.

Under current climatic conditions, its potential distribution has expanded markedly, particularly across central, southwestern, and northwestern China, as well as in the eastern and southern coastal regions, consistent with its affinity for temperate and humid monsoon climates (Tiansawat et al., 2022). However, future projections under high-emission scenarios (e.g., SSP585) suggest substantial habitat loss, likely driven by temperatures exceeding the physiological tolerance of species, coupled with terrain and anthropogenic constraints (Gao et al., 2024). By the 2050s and 2090s, regions such as Guangxi and Shandong are projected to lose more than 50% of their suitable habitat, with the remaining areas shifting to higher elevations. On the contrary, climate warming may facilitate range expansion into new regions, including Beijing, Hebei, Liaoning, Ningxia, and Heilongjiang, indicating a pronounced northward shift in distribution. Hunan Province is expected to retain the largest and most climatically suitable habitats, owing to its stable hydrothermal conditions.

### 4.4 Conservation strategies and research implications for *H. dulcis*

To mitigate the adverse impacts of climate warming on the distribution of *H. dulcis*, it is necessary to formulate an integrated conservation strategy that combines habitat protection, climate adaptation, and public participation. In regions with extensive suitable habitats, such as Hunan Province, establishing nature reserves or ecological corridors is crucial to protect existing populations and habitats. At the same time, strengthening the monitoring and management of hydrological and thermal systems is essential to ensure environmental stability. In areas experiencing substantial habitat reduction, including Guangxi Zhuang Autonomous Region and Shandong Province, local intervention measures such as artificial irrigation and shading should be implemented to alleviate heat and drought stress by optimizing microclimates. Meanwhile, proactive efforts should aim to expand the species’ range by establishing populations in newly identified suitable habitats in Beijing, Hebei, Liaoning, Ningxia, and Heilongjiang through artificial propagation and *ex situ* conservation. By taking advantage of the phenotypic plasticity of species at different environmental gradients and their inherent adaptability to diverse climatic zones, climate-adaptive breeding programs can focus on enhancing heat and drought tolerance to improve resilience in vulnerable regions (Nicotra et al., 2010). Furthermore, future studies should incorporate non-climatic factors, such as human interference and land use change, to improve the predictive models and develop effective and scientific management strategies, so as to preserve *H. dulcis* for a long time under changing environmental conditions.

## 5 Conclusion

This study employed a species distribution model to evaluate the impacts of climate change on the habitat suitability of *Hovenia dulcis* across China. The MaxEnt model demonstrated a high predictive accuracy (AUC = 0.934), and the species distribution is primarily affected by annual precipitation (Bio12), minimum temperature of the coldest month (Bio06), elevation, and mean diurnal temperature range (Bio02). Among them, annual precipitation (Bio12) and minimum temperature of the coldest month (Bio06) were the most influential, each contributing over 30% by percentage contribution and exceeding 16% by permutation importance, followed by elevation and diurnal temperature range. The results indicate that *H. dulcis* favors warm, humid subtropical monsoon climates, with optimal suitability occurring in mid-elevation hills of central, eastern, and southwestern China. Under future climate scenarios, its suitable range is expected to shift northward and upward in elevation. While regions such as Hunan, Jiangxi, Sichuan and Guizhou remain core suitable areas, northern provinces including Hebei, Liaoning, and Beijing are expected to become increasingly suitable. In contrast, habitats in Guangxi and Shandong may shrink significantly. These predicted shifts reflect the species’ dependence on warm temperatures and adequate moisture, highlighting its vulnerability and adaptive potential under global warming. These findings provide a scientific foundation for targeted conservation and sustainable utilization of *H. dulcis*. Priority measures should include *in situ* protection of core habitats, assisted migration into new suitable regions, and *ex situ* conservation combined with breeding programs focused on climate-adaptive traits.

## Data Availability

The original contributions presented in the study are included in the article/**Supplementary Material**. Further inquiries can be directed to the corresponding author.

## Supplementary material

Supplementary Table 1 Distribution information of all *H. dulcis* sample points collected in this study (n = 479).

Supplementary Table 2 Distribution information of *H. dulcis* after excluding samples with unclear information (n = 254).

Supplementary Table 3 Distribution information of *H. dulcis* based on 191 valid occurrence points.

Supplementary Table 4 Area of different suitable habitat types in Chinese provinces.

Supplementary Table 5 Total suitable habitat area in various provinces of China across different periods (sum of medium and high suitable habitat areas).

Supplementary Table 6 Change rate of suitable habitats in some provinces and observed values of different meteorological factors.

## References

[B1] Ahmadi M. Hemami M.-R. Kaboli M. Shabani F. (2023). MaxEnt brings comparable results when the input data are being completed; Model parameterization of four species distribution models. Ecol. Evol. 13, e9827. doi: 10.1002/ece3.9827, PMID: 36820245 PMC9937880

[B2] Aligaz M. A. Kufa C. A. Ahmed A. S. Argaw H. T. Tamrat M. Yihune M.. (2024). Distribution and extent of suitable habitats of Ruspoli’s Turaco (*Tauraco ruspolii*) and White-cheeked Turaco (*Tauraco leucotis*) under a changing climate in Ethiopia. BMC Ecol. Evol. 24, 83. doi: 10.1186/s12862-024-02245-y, PMID: 38902600 PMC11191209

[B3] Bergamin R. S. Gama M. Almerão M. Hofmann G. S. Anastácio P. M. (2022). Predicting current and future distribution of *Hovenia dulcis* Thunb. (Rhamnaceae) worldwide. Biol. Invasions 24, 2229–2243. doi: 10.1007/s10530-022-02771-0

[B4] Berman A. L. Silvestri G. E. Tonello M. S. (2018). On the differences between Last Glacial Maximum and Mid-Holocene climates in southern South America simulated by PMIP3 models. Quat. Sci. Rev. 185, 113–121. doi: 10.1016/j.quascirev.2018.02.003

[B5] Cao Z. Zhang L. Zhang X. Guo Z. (2021). Predicting the Potential Distribution of *Hylomecon japonica* in China under Current and Future Climate Change Based on Maxent Model. Sustainability 13, 11253. doi: 10.3390/su132011253

[B6] De Godoi R. S. Almerão M. P. Da Silva F. R. (2021). In silico evaluation of the antidiabetic activity of natural compounds from *Hovenia dulcis* Thunberg. J. Herb. Med. 28, 100349. doi: 10.1016/j.hermed.2020.100349

[B7] Duan Y. Bai H. Du Z. Liu Y. Li L. Lu K.. (2025). Maxent modeling for predicting the potential distribution of *Hippophae Linn* species. Trop. Ecol. 66, 132–145. doi: 10.1007/s42965-025-00372-1

[B8] Elith J. Phillips S. J. Hastie T. Dudík M. Chee Y. E. Yates C. J. (2011). A statistical explanation of MaxEnt for ecologists. Divers. Distrib. 17, 43–57. doi: 10.1111/j.1472-4642.2010.00725.x

[B9] Fan J. Li J. Xia R. Hu L. Wu X. Li G. (2014). Assessing the impact of climate change on the habitat distribution of the giant panda in the Qinling Mountains of China. Ecol. Model. 274, 12–20. doi: 10.1016/j.ecolmodel.2013.11.023

[B10] Gao H. Wei X. Peng Y. Zhuo Z. (2024). Predicting the impact of climate change on the future distribution of *Paederus fuscipes* Curtis 1826, in China based on the maxEnt model. Insects 15, 437. doi: 10.3390/insects15060437, PMID: 38921152 PMC11203407

[B11] Guisan A. Thuiller W. (2005). Predicting species distribution: offering more than simple habitat models. Ecol. Lett. 8, 993–1009. doi: 10.1111/j.1461-0248.2005.00792.x, PMID: 34517687

[B12] Guo T. Yang Q. Chen D. Wang X. Cheng Q. Wang S.. (2025). Assessment of Chinese suitable habitats of Amomum tsao-ko in different climatic conditions. Front. Plant Sci. 16. doi: 10.3389/fpls.2025.1561026, PMID: 40406721 PMC12095335

[B13] He Y. Liu M. Wang Y. Wu H. Wei M. Xue J.. (2024). *Hovenia dulcis*: a Chinese medicine that plays an essential role in alcohol-associated liver disease. Front. Pharmacol. 15. doi: 10.3389/fphar.2024.1337633, PMID: 38650630 PMC11033337

[B14] Hong S. H. Lee Y. H. Lee G. Lee D.-H. Adhikari P. (2021). Predicting impacts of climate change on northward range expansion of invasive weeds in South Korea. Plants Basel Switz. 10, 1604. doi: 10.3390/plants10081604, PMID: 34451649 PMC8401637

[B15] Huang B. Chen S. Xu L. Jiang H. Chen X. He H.. (2024). Predicting the potential geographical distribution of *Zingiber striolatum* Diels (Zingiberaceae), a medicine food homology plant in China. Sci. Rep. 14, 22206. doi: 10.1038/s41598-024-73202-4, PMID: 39333747 PMC11436980

[B16] Hyun T. Eom S. Yu C. Roitsch T. (2010). *Hovenia dulcis* – an Asian traditional herb. Planta Med. 76, 943–949. doi: 10.1055/s-0030-1249776, PMID: 20379955

[B17] Karuppaiah V. Maruthadurai R. Das B. Soumia P. S. Gadge A. S. Thangasamy A.. (2023). Predicting the potential geographical distribution of onion thrips, *Thrips tabaci* in India based on climate change projections using MaxEnt. Sci. Rep. 13, 7934. doi: 10.1038/s41598-023-35012-y, PMID: 37193780 PMC10188569

[B18] Khan A. M. Li Q. Saqib Z. Khan N. Habib T. Khalid N.. (2022). MaxEnt modelling and impact of climate change on habitat suitability variations of economically important chilgoza pine (*Pinus gerardiana* wall.) in south Asia. Forests 13, 715. doi: 10.3390/f13050715

[B19] Kumar D. Pandey A. Rawat S. Joshi M. Bajpai R. Upreti D. K.. (2022). Predicting the distributional range shifts of *Rhizocarpon geographicum* (L.) DC. @ in Indian Himalayan Region under future climate scenarios. Environ. Sci. pollut. Res. 29, 61579–61593. doi: 10.1007/s11356-021-15624-5, PMID: 34351582

[B20] Li S. Li Y. Hu M. Li Y. Yang M. Wang S.. (2025). Ecological risk assessment of future suitable areas for *Piper kadsura* under the background of climate change. Front. Plant Sci. 15. doi: 10.3389/fpls.2024.1471706, PMID: 39902198 PMC11788358

[B21] Li F. Park Y.-S. (2020). Habitat availability and environmental preference drive species range shifts in concordance with climate change. Divers. Distrib. 26, 1343–1356. doi: 10.1111/ddi.13126

[B22] Li M. Xie C. Meng C. Zhang Y. Gao J. Wang W.. (2021). Chemical constituents from *Hovenia dulcis* Thunb. and their chemotaxonomic significance. Biochem. Syst. Ecol. 94, 104214. doi: 10.1016/j.bse.2020.104214

[B23] Ma C. Xu X. Zhou M. Hu T. Qi C. (2024). A deep learning approach for chromium detection and characterization from soil hyperspectral data. Toxics 12, 357. doi: 10.3390/toxics12050357, PMID: 38787136 PMC11125944

[B24] Mahmoud A. R. Farahat E. A. Hassan L. M. Halmy M. W. A. (2025). Remotely sensed data contribution in predicting the distribution of native Mediterranean species. Sci. Rep. 15, 12475. doi: 10.1038/s41598-025-94569-y, PMID: 40216846 PMC11992134

[B25] Mao X. Zheng H. Luo G. Liao S. Wang R. Tang M.. (2024). Climate change favors expansion of three Eucalyptus species in China. Front. Plant Sci. 15. doi: 10.3389/fpls.2024.1443134, PMID: 39464280 PMC11502323

[B26] Melo-Merino S. M. Reyes-Bonilla H. Lira-Noriega A. (2020). Ecological niche models and species distribution models in marine environments: A literature review and spatial analysis of evidence. Ecol. Model. 415, 108837. doi: 10.1016/j.ecolmodel.2019.108837

[B27] Meng X. Tang G. Zhao C. Liu Q. Xu X. Cao S. (2020). Hepatoprotective effects of *Hovenia dulcis* seeds against alcoholic liver injury and related mechanisms investigated via network pharmacology. World J. Gastroenterol. 26, 3432–3446. doi: 10.3748/wjg.v26.i24.3432, PMID: 32655267 PMC7327782

[B28] Nicotra A. B. Atkin O. K. Bonser S. P. Davidson A. M. Finnegan E. J. Mathesius U.. (2010). Plant phenotypic plasticity in a changing climate. Trends Plant Sci. 15, 684–692. doi: 10.1016/j.tplants.2010.09.008, PMID: 20970368

[B29] Niiya M. Shimato Y. Ohno T. Makino T. (2024). Effects of *Hovenia dulcis* fruit and peduncle extract on alcohol metabolism. J. Ethnopharmacol. 321, 117541. doi: 10.1016/j.jep.2023.117541, PMID: 38052412

[B30] Ottó B. Végvári Z. (2022). Bioclimatic preferences of the great bustard in a steppe region. Diversity 14, 1138. doi: 10.3390/d14121138

[B31] Ouyang X. Pan J. Wu Z. Chen A. (2022). Predicting the potential distribution of *Campsis grandiflora* in China under climate change. Environ. Sci. pollut. Res. 29, 63629–63639. doi: 10.1007/s11356-022-20256-4, PMID: 35461417

[B32] Phillips S. J. Anderson R. P. Schapire R. E. (2006). Maximum entropy modeling of species geographic distributions. Ecol. Model. 190, 231–259. doi: 10.1016/j.ecolmodel.2005.03.026

[B33] Phillips S. J. Dudík M. (2008). Modeling of species distributions with Maxent: new extensions and a comprehensive evaluation. Ecography 31, 161–175. doi: 10.1111/j.0906-7590.2008.5203.x

[B34] Pischl P. H. Burke S. V. Bach E. M. Duvall M. R. (2020). Plastome phylogenomics and phylogenetic diversity of endangered and threatened grassland species (Poaceae) in a North American tallgrass prairie. Ecol. Evol. 10, 7602–7615. doi: 10.1002/ece3.6484, PMID: 32760551 PMC7391303

[B35] Pokharel K. P. Ludwig T. Storch I. (2016). Predicting potential distribution of poorly known species with small database: the case of four-horned antelope *Tetracerus quadricornis* on the Indian subcontinent. Ecol. Evol. 6, 2297–2307. doi: 10.1002/ece3.2037, PMID: 27069584 PMC4782261

[B36] Porfirio L. L. Harris R. M. B. Lefroy E. C. Hugh S. Gould S. F. Lee G.. (2014). Improving the use of species distribution models in conservation planning and management under climate change. PLoS One 9, e113749. doi: 10.1371/journal.pone.0113749, PMID: 25420020 PMC4242662

[B37] Riahi K. van Vuuren D. P. Kriegler E. Edmonds J. O’Neill B. C. Fujimori S.. (2017). The Shared Socioeconomic Pathways and their energy, land use, and greenhouse gas emissions implications: An overview. Glob. Environ. Change 42, 153–168. doi: 10.1016/j.gloenvcha.2016.05.009

[B38] Rong W. Huang X. Hu S. Zhang X. Jiang P. Niu P.. (2024). Impacts of climate change on the habitat suitability and natural product accumulation of the medicinal plant *Sophora alopecuroides* L. Based on the maxEnt model. Plants 13, 1424. doi: 10.3390/plants13111424, PMID: 38891233 PMC11174999

[B39] Sferrazza G. Brusotti G. Zonfrillo M. Temporini C. Tengattini S. Bononi M.. (2021). *Hovenia dulcis* thumberg: phytochemistry, pharmacology, toxicology and regulatory framework for its use in the European union. Mol. Basel Switz. 26, 903. doi: 10.3390/molecules26040903, PMID: 33572099 PMC7914479

[B40] Shen L. Deng H. Zhang G. Ma A. Mo X. (2023). Effect of climate change on the potentially suitable distribution pattern of *Castanopsis hystrix* miq. in China. Plants 12, 717. doi: 10.3390/plants12040717, PMID: 36840065 PMC9966962

[B41] Son Y. Lee D. H. Park G. H. Jang J.-H. Kim J. A. Park Y.. (2023). Comparison of Growth Characteristics and Active Compounds of Cultivated *Hovenia dulcis* under Different Environments in South Korea. Diversity 15, 905. doi: 10.3390/d15080905

[B42] Syfert M. M. Smith M. J. Coomes D. A. (2013). The effects of sampling bias and model complexity on the predictive performance of MaxEnt species distribution models. PLoS One 8, e55158. doi: 10.1371/journal.pone.0055158, PMID: 23457462 PMC3573023

[B43] Tang D. Chen M. Huang X. Zhang G. Zeng L. Zhang G.. (2023). SRplot: A free online platform for data visualization and graphing. PLoS One 18, e0294236. doi: 10.1371/journal.pone.0294236, PMID: 37943830 PMC10635526

[B44] Thomas C. D. Cameron A. Green R. E. Bakkenes M. Beaumont L. J. Collingham Y. C.. (2004). Extinction risk from climate change. Nature 427, 145–148. doi: 10.1038/nature02121, PMID: 14712274

[B45] Tiansawat P. Elliott S. D. Wangpakapattanawong P. (2022). Climate niche modelling for mapping potential distributions of four framework tree species: implications for planning forest restoration in tropical and subtropical Asia. Forests 13, 993. doi: 10.3390/f13070993

[B46] Tomczyk M. Zovko-Končić M. Chrostek L. (2012). Phytotherapy of alcoholism. Nat. Prod. Commun. 7, 273–280. doi: 10.1177/1934578X1200700243, PMID: 22474979

[B47] Wiens J. A. Stralberg D. Jongsomjit D. Howell C. A. Snyder M. A. (2009). Niches, models, and climate change: assessing the assumptions and uncertainties. Proc. Natl. Acad. Sci. U.S.A. 106 Suppl 2, 19729–19736. doi: 10.1073/pnas.0901639106, PMID: 19822750 PMC2780938

[B48] Yan H. Feng L. Zhao Y. Feng L. Wu D. Zhu C. (2020). Prediction of the spatial distribution of *Alternanthera philoxeroides* in China based on ArcGIS and MaxEnt. Glob. Ecol. Conserv. 21, e00856. doi: 10.1016/j.gecco.2019.e00856

[B49] Yang J. Jiang X. Ma Y. Liu M. Shama Z. Li J.. (2024). Potential global distribution of *Setaria italica*, an important species for dryland agriculture in the context of climate change. PLoS One 19, e0301751. doi: 10.1371/journal.pone.0301751, PMID: 38626039 PMC11020860

[B50] Zhan P. Wang F. Xia P. Zhao G. Wei M. Wei F.. (2022). Assessment of suitable cultivation region for *Panax notoginseng* under different climatic conditions using MaxEnt model and high-performance liquid chromatography in China. Ind. Crops Prod. 176, 114416. doi: 10.1016/j.indcrop.2021.114416

[B51] Zhang Y. Chen S. Gao Y. Yang L. Yu H. (2023). Prediction of global potential suitable habitats of *Zanthoxylum bungeanum* Link et Otto based on MaxEnt model. Sci. Rep. 13, 4851. doi: 10.1038/s41598-023-29678-7, PMID: 36964182 PMC10038996

[B52] Zhang K. Liu H. Pan H. Shi W. Zhao Y. Li S.. (2020). Shifts in potential geographical distribution of *Pterocarya stenoptera* under climate change scenarios in China. Ecol. Evol. 10, 4828–4837. doi: 10.1002/ece3.6236, PMID: 32551064 PMC7297781

[B53] Zhang M. Liu Z. Yang Z. Shen H. Wang J. Wu X. (2024). Altitudinal variation in species diversity, distribution, and regeneration status of a secondary *Picea* forest in Guandi mountain, northern China. Forests 15, 771. doi: 10.3390/f15050771

[B54] Zheng T. Sun J. Shi X. Liu D. Sun B. Deng Y.. (2022). Evaluation of climate factors affecting the quality of red huajiao (*Zanthoxylum bungeanum* maxim.) based on UPLC-MS/MS and MaxEnt model. Food Chem. X 16, 100522. doi: 10.1016/j.fochx.2022.100522, PMID: 36519100 PMC9743291

[B55] Zhou Y. Newman C. Xie Z. Macdonald D. W. (2013). Peduncles elicit large-mammal endozoochory in a dry-fruited plant. Ann. Bot. 112, 85–93. doi: 10.1093/aob/mct096, PMID: 23644364 PMC3690987

